# Supplementary material for: Personality traits are consistently associated with blood mitochondrial DNA copy number estimated from genome sequences in two genetic cohort studies
Source: eLife. 2022 Dec 20;11:e77806. doi: 10.7554/eLife.77806 (PMC9767459; doi:10.7554/eLife.77806)
Supplement: Source code 1. [file elife-77806-code1.zip › 10-02-2022-RA-eLife-77806/eLife_analysis_Rcode.pdf]

```
#======================================================
#Analysis script for mtDNAcn and personality association
#======================================================


#======================================================
# 1 Multiple linear regression of mtDNAcn on Personality
#======================================================
# BLSA
#variables to be analysed
variables<- names(blsa_neopir_final_pts)[c(11,14,23:(ncol(blsa_neopir_final_pts)-9),78)]

w_wo_wbcp<-1:3 #analysis with and without wbc parameters as covariates

btotal <- NA
bse_total<-NA
for(analysis in w_wo_wbcp){
  count <- 1
  btemp <- matrix(nrow=length(variables),ncol=8,dimnames=list(variables,c("Trait","Coefficient","lowerCI","upperCI","t_value","P_Value","R_Squared","n")))
  b_se<-matrix(nrow=length(variables),ncol=4,dimnames=list(variables,c("Trait","Coefficient","se","std_coef")))

  for(trait in variables){
    ##get your IV - all the applicable trait data
    IV <- blsa_neopir_final_pts[[trait]][!is.na(blsa_neopir_final_pts[[trait]])]

    ##get your IV aka the mt copy number for each ID
    DV <- blsa_neopir_final_pts$ave_mt_copy_number_30k[!is.na(blsa_neopir_final_pts[[trait]])]
    ##Standardize DV
    DV <- (DV-mean(DV))/sd(DV)
    ##get Age covariate
    CVAge <- blsa_neopir_final_pts$age[!is.na(blsa_neopir_final_pts[[trait]])]
    ##get Sex covariate
    CVSex <- blsa_neopir_final_pts$sex[!is.na(blsa_neopir_final_pts[[trait]])]
    ##linreg w covariates
    CVCov <- blsa_neopir_final_pts$auto_coverage_30k[!is.na(blsa_neopir_final_pts[[trait]])]
    ##adjust for blood traits
    CVPNE <- blsa_neopir_final_pts$CLPERCENTNEUTROPHILS[!is.na(blsa_neopir_final_pts[[trait]])]
    CVPLY <- blsa_neopir_final_pts$CLPERCENTLYMPHOCYTES[!is.na(blsa_neopir_final_pts[[trait]])]
    CVPMO <- blsa_neopir_final_pts$CLPERCENTMONOCYTES[!is.na(blsa_neopir_final_pts[[trait]])]
    CVPEO <- blsa_neopir_final_pts$CLPERCENTEOSINOPHILS[!is.na(blsa_neopir_final_pts[[trait]])]
    CVWBC <- blsa_neopir_final_pts$CLWBC[!is.na(blsa_neopir_final_pts[[trait]])]
    CVPBA <- blsa_neopir_final_pts$CLPERCENTBASOPHILS[!is.na(blsa_neopir_final_pts[[trait]])]
    CVPLT <- blsa_neopir_final_pts$CLPLATELETS[!is.na(blsa_neopir_final_pts[[trait]])]
    n <- length(IV)- sum(is.na(IV))

    if(analysis == 1){
      linreg<-lm(DV ~ IV)
      btemp[count,1]<- trait
      btemp[count,2:8]<-c(summary(linreg)$coef[2,"Estimate"],confint(linreg,'IV',level = 0.95)[,1],confint(linreg,'IV',level = 0.95)[,2],summary(linreg)$coef[2,"t
 value"], summary(linreg)$coef[2,"Pr(>|t|)"], summary(linreg)$r.squared,n)
      b_se[count,1]<-trait
      b_se[count,2:4]<-c(summary(linreg)$coef[2,"Estimate"], summary(linreg)$coef[2,"Std. Error"],summary(linreg)$coef[2,"Estimate"]*sd(IV)/sd(DV))
    }
    else if(analysis == 2){
      if(trait=="age"){linreg <- lm(DV ~ IV  + CVSex + CVCov)}
      else if(trait=="sex"){linreg <- lm(DV ~ IV + CVAge + CVCov )}
      else if(trait=="auto_coverage_30k"){linreg <- lm(DV ~ IV + CVAge + CVSex )}
      else{linreg <- lm(DV ~ IV + CVAge + CVSex + CVCov )}
      btemp[count,1]<- trait
      btemp[count,2:8]<-c(summary(linreg)$coef[2,"Estimate"],confint(linreg,'IV',level = 0.95)[,1],confint(linreg,'IV',level = 0.95)[,2],summary(linreg)$coef[2,"t
 value"], summary(linreg)$coef[2,"Pr(>|t|)"], summary(linreg)$r.squared,n)
      b_se[count,1]<-trait
      b_se[count,2:4]<-c(summary(linreg)$coef[2,"Estimate"], summary(linreg)$coef[2,"Std. Error"],summary(linreg)$coef[2,"Estimate"]*sd(IV)/sd(DV))
    }

    else{
      if(trait=="age"){linreg <- lm(DV ~ IV  + CVSex + CVCov + CVPNE + CVPLY + CVPMO + CVPEO + CVWBC+CVPBA+CVPLT)}
      else if(trait=="sex"){linreg <- lm(DV ~ IV + CVAge + CVCov + CVPNE + CVPLY + CVPMO + CVPEO + CVWBC+CVPBA+CVPLT)}
      else if(trait=="CLPERCENTNEUTROPHILS"){linreg <- lm(DV ~ IV + CVAge + CVSex + CVCov + CVPLY + CVPMO + CVPEO + CVWBC+CVPBA+CVPLT)}
      else if(trait=="CLPERCENTLYMPHOCYTES"){linreg <- lm(DV ~ IV + CVAge + CVSex + CVCov + CVPNE + CVPMO + CVPEO + CVWBC+CVPBA+CVPLT)}
      else if(trait=="CLPERCENTMONOCYTES"){linreg <- lm(DV ~ IV + CVAge + CVSex + CVCov + CVPNE + CVPLY + CVPEO + CVWBC+CVPBA+CVPLT)}
      else if(trait=="CLPERCENTEOSINOPHILS"){linreg <- lm(DV ~ IV + CVAge + CVSex + CVCov + CVPNE + CVPLY + CVPMO + CVWBC+CVPBA+CVPLT)}
      else if(trait=="CLPERCENTBASOPHILS"){linreg <- lm(DV ~ IV + CVAge + CVSex + CVCov + CVPNE + CVPLY + CVPMO + CVWBC+CVPEO+CVPLT)}
      else if(trait=="CLWBC"){linreg <- lm(DV ~ IV + CVAge + CVSex + CVCov + CVPNE + CVPLY + CVPMO + CVPEO+CVPBA+CVPLT)}
      else if(trait=="CLPLATELETS"){linreg <- lm(DV ~ IV + CVAge + CVSex + CVCov + CVPNE + CVPLY + CVPMO + CVPEO+CVPBA+CVWBC)}

      else{linreg <- lm(DV ~ IV + CVAge + CVSex + CVCov + CVPNE + CVPLY + CVPMO + CVPEO + CVWBC+CVPBA+CVPLT)}
      btemp[count,1]<- trait
      btemp[count,2:8]<-c(summary(linreg)$coef[2,"Estimate"],confint(linreg,'IV',level = 0.95)[,1],confint(linreg,'IV',level = 0.95)[,2],summary(linreg)$coef[2,"t
 value"], summary(linreg)$coef[2,"Pr(>|t|)"], summary(linreg)$r.squared,n)
      b_se[count,1]<-trait
      b_se[count,2:4]<-c(summary(linreg)$coef[2,"Estimate"], summary(linreg)$coef[2,"Std. Error"],summary(linreg)$coef[2,"Estimate"]*sd(IV)/sd(DV))
    }
    count <- count+1
  }
  if(analysis==1){btemp1<-as.data.frame(btemp)}
  if(analysis==2){btemp2<-as.data.frame(btemp)}
  if(analysis==3){btemp3<-as.data.frame(btemp)}
  if(analysis==1){b_se1<-as.data.frame(b_se)}
  if(analysis==2){b_se2<-as.data.frame(b_se)}
  if(analysis==3){b_se3<-as.data.frame(b_se)}
  rm(btemp)
  rm(b_se)
}

btemp1[,6]<-as.numeric(as.character(btemp1[,6]))
btemp2[,6]<-as.numeric(as.character(btemp2[,6]))
btemp3[,6]<-as.numeric(as.character(btemp3[,6]))
```

```
btemp1 <- btemp1[order(btemp1[,6]),]
btemp2 <- btemp2[order(btemp2[,6]),]
btemp3 <- btemp3[order(btemp3[,6]),]

btotal<-cbind(btemp1,btemp2,btemp3)
write.csv(btotal, file="BLSA_final_analysis_results_mtdnacnstd.csv",row.names=F)
bse_total<-cbind(b_se1,b_se2,b_se3)
write.csv(bse_total, file="BLSA_final_analysis_se_mtdnacnstd.csv",row.names=F)


#------------------
# Sardinia analysis
#------------------
##pick traits to analyze
traits <- names(wavedata1_plt)[c(6:7,12:88,117)]
MinRealPoints <- 25 ##Amount of real points needed to be considered for being analyzed - can be made less stringent

stotal <- NA
std_total<-NA
for(analysis in w_wo_wbcp){
  count <- 1
  stemp <- matrix(nrow=length(traits),ncol=8,dimnames=list(traits,c("Trait","Coefficient","lowerCI","upperCI","t_value","P_Value","R_Squared","n")))
  std_err<-matrix(nrow=length(traits),ncol=4,dimnames=list(traits,c("Trait","Coefficient","se","std_coef_b")))
  for(trait in traits){
    ##get your IV - all the applicable trait data
    IV <- wavedata1_plt[[trait]][!is.na(wavedata1_plt[[trait]])]

    ##get your DV aka the mt copy number for each ID
    DV <- wavedata1_plt$MT_count[!is.na(wavedata1_plt[[trait]])]
    #standardise DV
    DV <- (DV-mean(DV))/sd(DV)
    ##get Age covariat
    CVAge <- wavedata1_plt$Age[!is.na(wavedata1_plt[[trait]])]
    ##get Sex covariat
    CVSex <- wavedata1_plt$Sex[!is.na(wavedata1_plt[[trait]])]
    ##linreg w covariats
    CVCov <- wavedata1_plt$Chrom_Coverage[!is.na(wavedata1_plt[[trait]])]
    ##adjust for blood traits
    CVPNE <- wavedata1_plt$labsPercNE[!is.na(wavedata1_plt[[trait]])]
    CVPLY <- wavedata1_plt$labsPercLY[!is.na(wavedata1_plt[[trait]])]
    CVPMO <- wavedata1_plt$labsPercMO[!is.na(wavedata1_plt[[trait]])]
    CVPEO <- wavedata1_plt$labsPercEO[!is.na(wavedata1_plt[[trait]])]
    CVPBA <- wavedata1_plt$labsPercBA[!is.na(wavedata1_plt[[trait]])]
    CVWBC <- wavedata1_plt$labsWBC[!is.na(wavedata1_plt[[trait]])]
    CVPLT <- wavedata1_plt$labsPLT[!is.na(wavedata1_plt[[trait]])]
    n <- length(IV)- sum(is.na(IV))
    if(MinRealPoints <= n){

      if(analysis == 1){
        linreg<-lm(DV ~ IV)
        stemp[count,1]<- trait

        stemp[count,2:8]<-c(summary(linreg)$coef[2,"Estimate"],confint(linreg,'IV',level = 0.95)[,1],confint(linreg,'IV',level = 0.95)[,2],summary(linreg)$coef[2,"t
value"], summary(linreg)$coef[2,"Pr(>|t|)"], summary(linreg)$r.squared,n)
        std_err[count,1]<-trait
        std_err[count,2:4]<-c(summary(linreg)$coef[2,"Estimate"], summary(linreg)$coef[2,"Std. Error"],summary(linreg)$coef[2,"Estimate"]*sd(IV)/sd(DV))
      }

      else if(analysis == 2){
        if(trait=="Age"){linreg <- lm(DV ~ IV  + CVSex + CVCov)}
        else if(trait=="Sex"){linreg <- lm(DV ~ IV + CVAge + CVCov )}
        else if(trait=="Chrom_Coverage"){linreg <- lm(DV ~ IV + CVAge + CVSex )}
        else{linreg <- lm(DV ~ IV + CVAge + CVSex + CVCov )}
        stemp[count,1]<- trait
        stemp[count,2:8]<-c(summary(linreg)$coef[2,"Estimate"],confint(linreg,'IV',level = 0.95)[,1],confint(linreg,'IV',level = 0.95)[,2],summary(linreg)$coef[2,"t
value"], summary(linreg)$coef[2,"Pr(>|t|)"], summary(linreg)$r.squared,n)
        std_err[count,1]<-trait
        std_err[count,2:4]<-c(summary(linreg)$coef[2,"Estimate"], summary(linreg)$coef[2,"Std. Error"],summary(linreg)$coef[2,"Estimate"]*sd(IV)/sd(DV))
      }
      else {
        if(trait=="Age"){linreg <- lm(DV ~ IV  + CVSex + CVCov + CVPNE + CVPLY + CVPMO + CVPEO + CVPBA + CVWBC+CVPLT)}
        else if(trait=="Sex"){linreg <- lm(DV ~ IV + CVAge + CVCov + CVPNE + CVPLY + CVPMO + CVPEO + CVPBA + CVWBC+CVPLT)}
        else if(trait=="labsPercNE"){linreg <- lm(DV ~ IV + CVAge + CVSex + CVCov + CVPLY + CVPMO + CVPEO + CVPBA+ CVWBC+CVPLT)}
        else if(trait=="labsPercLY"){linreg <- lm(DV ~ IV + CVAge + CVSex + CVCov + CVPNE + CVPMO + CVPEO + CVPBA + CVWBC+CVPLT)}
        else if(trait=="labsPercMO"){linreg <- lm(DV ~ IV + CVAge + CVSex + CVCov + CVPNE + CVPLY + CVPEO + CVPBA + CVWBC+CVPLT)}
        else if(trait=="labsPercEO"){linreg <- lm(DV ~ IV + CVAge + CVSex + CVCov + CVPNE + CVPLY + CVPMO + CVPBA + CVWBC+CVPLT)}
        else if(trait=="labsPercBA"){linreg <- lm(DV ~ IV + CVAge + CVSex + CVCov + CVPNE + CVPLY + CVPMO + CVPEO+ CVWBC+CVPLT)}
        else if(trait=="labsWBC"){linreg <- lm(DV ~ IV + CVAge + CVSex + CVCov + CVPNE + CVPLY + CVPMO + CVPEO + CVPBA+CVPLT)}
        else if(trait=="labsPLT"){linreg <- lm(DV ~ IV + CVAge + CVSex + CVCov + CVPNE + CVPLY + CVPMO + CVPEO + CVPBA+CVWBC)}

        else{linreg <- lm(DV ~ IV + CVAge + CVSex + CVCov + CVPNE + CVPLY + CVPMO + CVPEO + CVPBA + CVWBC+CVPLT)}
        stemp[count,1]<- trait
        stemp[count,2:8]<-c(summary(linreg)$coef[2,"Estimate"],confint(linreg,'IV',level = 0.95)[,1],confint(linreg,'IV',level = 0.95)[,2],summary(linreg)$coef[2,"t
value"], summary(linreg)$coef[2,"Pr(>|t|)"], summary(linreg)$r.squared,n)
        std_err[count,1]<-trait
        std_err[count,2:4]<-c(summary(linreg)$coef[2,"Estimate"], summary(linreg)$coef[2,"Std. Error"],summary(linreg)$coef[2,"Estimate"]*sd(IV)/sd(DV))
      }}
    else{
      stemp[count,]<-NA
      std_err[count,]<-NA
    }
    count <- count+1

  }
  if(analysis==1){stemp1<-as.data.frame(stemp)}
  if(analysis==2){stemp2<-as.data.frame(stemp)}
  if(analysis==3){stemp3<-as.data.frame(stemp)}
  if(analysis==1){std_err1<-as.data.frame(std_err)}
```

```r
  if(analysis==2){std_err2<-as.data.frame(std_err)}
  if(analysis==3){std_err3<-as.data.frame(std_err)}

  rm(stemp)
  rm(std_err)
}
stemp1[,6]<-as.numeric(as.character(stemp1[,6]))
stemp2[,6]<-as.numeric(as.character(stemp2[,6]))
stemp3[,6]<-as.numeric(as.character(stemp3[,6]))


stemp1 <- stemp1[order(stemp1[,6]),]
stemp2 <- stemp2[order(stemp2[,6]),]
stemp3 <- stemp3[order(stemp3[,6]),]
stotal<-cbind(stemp1,stemp2,stemp3)
write.csv(stotal, file="Sardinia_final_analysis_results_mtdnacnstd.csv",row.names=F)
std_total<-cbind(std_err1,std_err2,std_err3)
write.csv(std_total, file="Sardinia_final_analysis_se_mtdnacnstd.csv",row.names=F)


#=========================================================================
# 2 Linear Regression of mtDNAcn on CES-D (measure of depressive symptoms)
#=========================================================================

### BLSA #######
#standardize variables
blsa_mtdna_cesd_plt$ave_mt_copy_number_30k_std<-(blsa_mtdna_cesd_plt$ave_mt_copy_number_30k-mean(blsa_mtdna_cesd_plt$ave_mt_copy_number_30k))/
 sd(blsa_mtdna_cesd_plt$ave_mt_copy_number_30k)

blsa_mtdna_cesd_plt$CEScut16<-as.factor(blsa_mtdna_cesd_plt$CEScut16)
blsa_mtdna_cesd_plt$CEScut20<-as.factor(blsa_mtdna_cesd_plt$CEScut20)
fitcesd<-lm(ave_mt_copy_number_30k_std~CESD,data = blsa_mtdna_cesd_plt)
fitcesd16<-lm(ave_mt_copy_number_30k_std~CEScut16,data = blsa_mtdna_cesd_plt)
fitcesd20<-lm(ave_mt_copy_number_30k_std~CEScut20,data = blsa_mtdna_cesd_plt)

## Adjusted for age, sex and coverage
fitcesd<-lm(ave_mt_copy_number_30k_std~age+sex+auto_coverage_30k+CESD,data = blsa_mtdna_cesd_plt)
fitcesd16<-lm(ave_mt_copy_number_30k_std~age+sex+auto_coverage_30k+CEScut16,data = blsa_mtdna_cesd_plt)
fitcesd20<-lm(ave_mt_copy_number_30k_std~age+sex+auto_coverage_30k+CEScut20,data = blsa_mtdna_cesd_plt)

# adjusting for age sex coverage and wbc parameters
fitcesd<-lm(ave_mt_copy_number_30k_std~age+sex+
              auto_coverage_30k+CLWBC+CLPERCENTLYMPHOCYTES+CLPERCENTEOSINOPHILS+
              CLPERCENTMONOCYTES+CLPERCENTNEUTROPHILS+CLPERCENTBASOPHILS+CLPLATELETS+CESD,data = blsa_mtdna_cesd_plt)
fitcesd16<-lm(ave_mt_copy_number_30k_std~age+sex+
                auto_coverage_30k+CLWBC+CLPERCENTLYMPHOCYTES+CLPERCENTEOSINOPHILS+
                CLPERCENTMONOCYTES+CLPERCENTNEUTROPHILS+CLPERCENTBASOPHILS+CLPLATELETS+CEScut16,data = blsa_mtdna_cesd_plt)
fitcesd20<-lm(ave_mt_copy_number_30k_std~age+sex+
                auto_coverage_30k+CLWBC+CLPERCENTLYMPHOCYTES+CLPERCENTEOSINOPHILS+
                CLPERCENTMONOCYTES+CLPERCENTNEUTROPHILS+CLPERCENTBASOPHILS+CLPLATELETS+CEScut20,data = blsa_mtdna_cesd_plt)

####SardiNIA#######
#standardize variables
wavedata3_plt$MT_count_std<-(wavedata3_plt$MT_count-mean(wavedata3_plt$MT_count,na.rm = T))/sd(wavedata3_plt$MT_count,na.rm = T)
fitcesd<-lm(MT_count_std~CESD,data = wavedata3_plt)
fitcesd16<-lm(MT_count_std~CESDcut16,data = wavedata3_plt)
fitcesd20<-lm(MT_count_std~CESDcut20,data = wavedata3_plt)

## Adjusted for age, sex and coverage
fitcesd<-lm(MT_count_std~Age+Sex+Chrom_Coverage+CESD,data = wavedata3_plt)
wavedata3_plt$CESDcut16<-as.factor(wavedata3_plt$CESDcut16)
wavedata3_plt$CESDcut20<-as.factor(wavedata3_plt$CESDcut20)
fitcesd16<-lm(MT_count_std~Age+Sex+Chrom_Coverage+CESDcut16,data = wavedata3_plt)
fitcesd20<-lm(MT_count_std~Age+Sex+Chrom_Coverage+CESDcut20,data = wavedata3_plt)

# adjusting for age sex coverage and wbc parameters
fitcesd<-lm(MT_count_std~Age+Chrom_Coverage+labsWBC+labsPercNE+labsPercEO+
              labsPercLY+labsPercMO+Sex+ labsPLT+CESD,data = wavedata3_plt)
fitcesd16<-lm(MT_count_std~Age+Chrom_Coverage+labsWBC+labsPercNE+
                labsPercEO+labsPercLY+labsPercMO+Sex+labsPLT+CESDcut16,data = wavedata3_plt)
fitcesd20<-lm(MT_count_std~Age+Chrom_Coverage+labsWBC+labsPercNE+
                labsPercEO+labsPercLY+labsPercMO+Sex+ labsPLT+CESDcut20,data = wavedata3_plt)


#===========================================================================
# 3 Personality mortality Index
#===========================================================================
## Vulnerability, Activity, Competence and Self discipline facets
#We propose that we could use a list of traits that are associated with mortality risk
#in Chapman et al. 2020 and assign a score of 0 or 1 to each individual based on the median value
#and the direction of the association of the trait. Then we can get a composite score for each
#individual by summing up the individual trait scores (creating an index)


#======BLSA=================
median(blsa_neopir_final_pts$neotn6,na.rm = T) #median of vulnerability 46.15385
median(blsa_neopir_final_pts$neotc5,na.rm = T) #median of self discipline 50.07752
median(blsa_neopir_final_pts$neote4,na.rm = T) #median of activity 51.32231
median(blsa_neopir_final_pts$neotc1,na.rm = T) #median of competence 54.73469

# create column of 4 distinct personality types
blsa_neopir_final_pts$vu<-NA  #vulnerability
#high vulnerability is 0
blsa_neopir_final_pts$vu[which(blsa_neopir_final_pts$neotn6>46.15385)]<-0
blsa_neopir_final_pts$vu[which(blsa_neopir_final_pts$neotn6<=46.15385)]<-1

blsa_neopir_final_pts$sd<-NA  #self discipline
#high self discipline is 1
blsa_neopir_final_pts$sd[which(blsa_neopir_final_pts$neotc5>50.07752)]<-1
```

```
blsa_neopir_final_pts$sd[which(blsa_neopir_final_pts$neotc5<=50.07752)]<-0

blsa_neopir_final_pts$ac<-NA  #activity
#high activity is 1
blsa_neopir_final_pts$ac[which(blsa_neopir_final_pts$neote4>51.32231)]<-1
blsa_neopir_final_pts$ac[which(blsa_neopir_final_pts$neote4<=51.32231)]<-0

blsa_neopir_final_pts$co<-NA  #competence
#high competence is 1
blsa_neopir_final_pts$co[which(blsa_neopir_final_pts$neotc1>54.73469)]<-1
blsa_neopir_final_pts$co[which(blsa_neopir_final_pts$neotc1<=54.73469)]<-0

#Summing to create Composite trait
blsa_neopir_final_pts$mort_comp<-blsa_neopir_final_pts$vu +blsa_neopir_final_pts$sd + blsa_neopir_final_pts$ac  +blsa_neopir_final_pts$co

#box plots
library(dplyr)
library(ggplot2)
library(grid)
library(gridExtra)
library(ggpubr)

blsa_neopir_final_pts$mort_compf<-factor(blsa_neopir_final_pts$mort_comp,levels = sort(unique(blsa_neopir_final_pts$mort_comp)))
my_comp_m<-list(c('0','1'),c('0','2'),c('0','3'),c('0','4'))

mort_plot<-ggboxplot(data=subset(blsa_neopir_final_pts,!is.na(mort_compf)),x='mort_compf',y='std_mtdnacn',color='mort_compf',palette = 'jco',add = 'jitter',legend='none')
mort_plot<-mort_plot+ggtitle('A                                                                    \n  ')
mort_plot<-mort_plot+annotate('text',x=4,y=5,label='p-value < 0.005',fontface=2)#+stat_compare_means(method = 'anova',label.y =
 3.5)+stat_compare_means(comparisons=my_comp_m, method = 't.test',label= 'p.format')#,label.y = 420)
mort_plot<-mort_plot+my_own_theme()+theme(legend.position='none',panel.grid = element_blank())+xlab('Personality-Mortality Indices')+ylab('Standardized mtDNA copy
 number')
mort_plot
ggsave(mort_plot, file="Pers_mort_BLSA_final_std.jpeg", width=7, height=7)

# t test of the composite groups
t.test(blsa_neopir_final_pts$std_mtdnacn[which(blsa_neopir_final_pts$mort_compf==0)],blsa_neopir_final_pts$std_mtdnacn[which(blsa_neopir_final_pts$mort_compf==1)])
t.test(blsa_neopir_final_pts$std_mtdnacn[which(blsa_neopir_final_pts$mort_compf==0)],blsa_neopir_final_pts$std_mtdnacn[which(blsa_neopir_final_pts$mort_compf==2)])
t.test(blsa_neopir_final_pts$std_mtdnacn[which(blsa_neopir_final_pts$mort_compf==0)],blsa_neopir_final_pts$std_mtdnacn[which(blsa_neopir_final_pts$mort_compf==3)])
t.test(blsa_neopir_final_pts$std_mtdnacn[which(blsa_neopir_final_pts$mort_compf==0)],blsa_neopir_final_pts$std_mtdnacn[which(blsa_neopir_final_pts$mort_compf==4)])

# effect on life
#with adjustments
summary(lm(age~wo_age_resid_std, data=blsa_neopir_final_pts))


#for vu_sd

blsa_neopir_final_pts$vu_sd<-factor(blsa_neopir_final_pts$vu_sd,levels = sort(unique(blsa_neopir_final_pts$vu_sd)))
my_comp<-list(c('LVHD','HVLD'),c('HVLD','HVHD'),c('HVLD','LVLD'))

vusd_plot<-ggboxplot(data=subset(blsa_neopir_final_pts,!is.na(vu_sd)),x='vu_sd',y='std_mtdnacn',color='vu_sd',palette = 'jco',add = 'jitter',legend='none')
vusd_plot<-vusd_plot+ggtitle('A                                                            \n  ')
vusd_plot<-vusd_plot+stat_compare_means(method = 'anova',label.y = 6)+stat_compare_means(comparisons=my_comp, method = 't.test',label= 'p.format')#,label.y = 420)
vusd_plot<-vusd_plot+my_own_theme()+theme(legend.position='none',panel.grid = element_blank())+xlab('Vulnerability and Self-discipline classes')+ylab('Standardized mtDNA
 copy number')
vusd_plot
ggsave(vusd_plot, file="VDmort_BLSA_final_std.jpeg", width=6, height=6)

# t test of highest and lowest mortality risk groups (HVLD and LVHD)
t.test(blsa_neopir_final_pts$std_mtdnacn[which(blsa_neopir_final_pts$vu_sd=='HVLD')],blsa_neopir_final_pts$std_mtdnacn[which(blsa_neopir_final_pts$vu_sd=='LVHD')])


#### SardiNIA #####
sard_morts_original<-sard_morts #created December 28 2020
sard_morts<-all_traits_unrelated[which(all_traits_unrelated$Wave==1),
                                 c('id_individual','MT_count','neotn6','neotc5','neote4','neotc1')]
median(sard_morts$neotn6,na.rm = T) #median of vulnerability 55.12821
median(sard_morts$neotc5,na.rm = T) #median of self discipline 50.46512
median(sard_morts$neote4,na.rm = T) #median of activity 50.90909
median(sard_morts$neotc1,na.rm = T) #median of competence 43.71429

# create column of 4 distinct personality types
sard_morts$vu<-NA  #vulnerability
#high vulnerability is 0
sard_morts$vu[which(sard_morts$neotn6>55.12821)]<-0
sard_morts$vu[which(sard_morts$neotn6<=55.12821)]<-1

sard_morts$sd<-NA  #self discipline
#high self discipline is 1
sard_morts$sd[which(sard_morts$neotc5>50.46512)]<-1
sard_morts$sd[which(sard_morts$neotc5<=50.46512)]<-0

sard_morts$ac<-NA  #activity
#high activity is 1
sard_morts$ac[which(sard_morts$neote4>50.90909)]<-1
sard_morts$ac[which(sard_morts$neote4<=50.90909)]<-0

sard_morts$co<-NA  #competence
#high competence is 1
sard_morts$co[which(sard_morts$neotc1>43.71429)]<-1
sard_morts$co[which(sard_morts$neotc1<=43.71429)]<-0

#Summing to create Composite trait
sard_morts$mort_comp<-sard_morts$vu +sard_morts$sd + sard_morts$ac  +sard_morts$co
sard_morts$std_MT_count<-(sard_morts$MT_count-mean(sard_morts$MT_count,na.rm = T))/sd(sard_morts$MT_count,na.rm = T)

#Plot
sard_morts$mort_compf<-factor(sard_morts$mort_comp,levels = sort(unique(sard_morts$mort_comp)))
my_comp_m<-list(c('0','1'),c('0','2'),c('0','3'),c('0','4'))
```

```r
mort_plot<-ggboxplot(data=subset(sard_morts,!is.na(mort_compf)),x='mort_compf',y='std_MT_count',color='mort_compf',palette = 'jco',add = 'jitter',legend='none')
mort_plot<-mort_plot+ggtitle('B                                                                 \n  ')
mort_plot<-mort_plot+annotate('text',x=4,y=5,label='p-value < 0.007',fontface=2)#+stat_compare_means(method = 'anova',label.y =
 3)+stat_compare_means(comparisons=my_comp_m, method = 't.test',label= 'p.format')#,label.y = 420)
mort_plot<-mort_plot+my_own_theme()+theme(legend.position='right',legend.title = element_text(size = 14,face = "bold"),panel.grid = element_blank())+xlab('Personality-
 Mortality Indices')+ylab('Standardized mtDNA copy number')+labs(color="PMI")
mort_plot
ggsave(mort_plot, file="Pers_mort_Sardinia_final_std2.jpeg", width=7, height=7)


# t test of highest and lowest mortality risk groups
t.test(sard_morts$std_MT_count[which(sard_morts$mort_compf==0)],sard_morts$std_MT_count[which(sard_morts$mort_compf==1)])
t.test(sard_morts$std_MT_count[which(sard_morts$mort_compf==0)],sard_morts$std_MT_count[which(sard_morts$mort_compf==2)])
t.test(sard_morts$std_MT_count[which(sard_morts$mort_compf==0)],sard_morts$std_MT_count[which(sard_morts$mort_compf==3)])
t.test(sard_morts$std_MT_count[which(sard_morts$mort_compf==0)],sard_morts$std_MT_count[which(sard_morts$mort_compf==4)])


#Difference equivalence in years
#with no adjustments
summary(lm(Age~std_MT_count, data=wavedata1_plt))

#with adjustments
summary(lm(Age~wo_age_resid_std, data=wavedata1_plt))

#for vu_sd
sard_traits$std_MT_count<-(sard_traits$MT_count-mean(sard_traits$MT_count,na.rm = T))/sd(sard_traits$MT_count,na.rm = T)

sard_traits$vu_sd<-factor(sard_traits$vu_sd,levels = sort(unique(sard_traits$vu_sd)))
my_comp<-list(c('LVHD','HVLD'),c('HVLD','HVHD'),c('HVLD','LVLD'))

vusd_plot<-ggboxplot(data=subset(sard_traits,!is.na(vu_sd)),x='vu_sd',y='std_MT_count',color='vu_sd',palette = 'jco',add = 'jitter',legend='none')
vusd_plot<-vusd_plot+ggtitle('B                                                    \n  ')
vusd_plot<-vusd_plot+stat_compare_means(method = 'anova',label.y = 5)+stat_compare_means(comparisons=my_comp, method = 't.test',label= 'p.format')#,label.y = 420)
vusd_plot<-vusd_plot+my_own_theme()+theme(legend.position='none',panel.grid = element_blank())+xlab('Vulnerability and Self-discipline classes')+ylab('Standardized mtDNA
 copy number')
vusd_plot
ggsave(vusd_plot, file="VDmort_SardiNIA_final_std.jpeg", width=6, height=6)

# t test of highest and lowest mortality risk groups (HVLD and LVHD)
t.test(sard_traits$std_MT_count[which(sard_traits$vu_sd=='HVLD')],sard_traits$std_MT_count[which(sard_traits$vu_sd=='LVHD')])


#==================================================
# 4 Random effects meta analysis using library meta
#==================================================
#library used is meta, functions used are metagen and forest
library(meta)
#CESD
meta_cesd_std<-metagen(TE=estimate,seTE = se,data = meta_analysis_final_std_plt[1:2,],studlab = paste(Study),comb.fixed = F,comb.random = T,hakn = F,prediction =
 T,sm='SMD')
meta_cesd_std
forest(meta_cesd_std,layout = 'JAMA',text.predict = '95% PI',col.predict = 'black',xlab = "Effect size (95% CI)",colgap.forest = '1cm',colgap.studlab = '1cm')

meta_cesdc_std<-metagen(TE=estimate,seTE = se,data = meta_analysis_final_std_plt[3:6,],studlab = paste(Study),comb.fixed = F,comb.random = T,hakn = F,prediction =
 T,sm='SMD')
meta_cesdg_std<-update.meta(meta_cesdc_std,byvar = Facet,comb.random = T,comb.fixed = F)
meta_cesdg_std
forest(meta_cesdg_std,layout = 'JAMA',text.predict = '95% PI',col.predict = 'black',xlab = "Effect size (95% CI)",colgap.forest = '1cm',colgap.studlab = '1cm')

#Personality Mortality Composite
meta_mort_std<-metagen(TE=estimate,seTE = se,data = meta_analysis_final_std_plt[7:8,],studlab = paste(Study),comb.fixed = F,comb.random = T,hakn = F,prediction =
 T,sm='SMD')
meta_mort_std
forest(meta_mort_std,layout = 'JAMA',text.predict = '95% PI',col.predict = 'black',xlab = "Effect size (95% CI)",colgap.forest = '1cm',colgap.studlab = '1cm')

#agreeableness
metr_a_std<-metagen(TE=estimate,seTE = se,data = meta_analysis_final_std_plt[9:22,],studlab = paste(Study),comb.fixed = F,comb.random = T,hakn = F,prediction =
 T,sm='SMD')
metr_ag_std<-update.meta(metr_a_std,byvar = Facet,comb.random = T,comb.fixed = F)
metr_ag_std
forest(metr_ag_std,layout = 'JAMA',text.predict = '95% PI',col.predict = 'black',xlab = "Effect size (95% CI)",colgap.forest = '1cm',colgap.studlab = '1cm')

#conscientiousness
metr_c_std<-metagen(TE=estimate,seTE = se,data = meta_analysis_final_std_plt[23:36,],studlab = paste(Study),comb.fixed = F,comb.random = T,hakn = F,prediction =
 T,sm='SMD')
metr_cg_std<-update.meta(metr_c_std,byvar = Facet,comb.random = T,comb.fixed = F)
metr_cg_std
forest(metr_cg_std,layout = 'JAMA',text.predict = '95% PI',col.predict = 'black',xlab = "Effect size (95% CI)",colgap.forest = '2.5cm',colgap.studlab = '1cm')

#extraversion
metr_e_std<-metagen(TE=estimate,seTE = se,data = meta_analysis_final_std_plt[37:50,],studlab = paste(Study),comb.fixed = F,comb.random = T,hakn = F,prediction =
 T,sm='SMD')
metr_eg_std<-update.meta(metr_e_std,byvar = Facet,comb.random = T,comb.fixed = F)
metr_eg_std
forest(metr_eg_std,layout = 'JAMA',text.predict = '95% PI',col.predict = 'black',xlab = "Effect size (95% CI)",colgap.forest = '1cm',colgap.studlab = '1cm')

#neuroticism
metr_n_std<-metagen(TE=estimate,seTE = se,data = meta_analysis_final_std_plt[51:64,],studlab = paste(Study),comb.fixed = F,comb.random = T,hakn = F,prediction =
 T,sm='SMD')
metr_ng_std<-update.meta(metr_n_std,byvar =Facet,comb.random = T,comb.fixed = F)
metr_ng_std
forest(metr_ng_std,layout = 'JAMA',text.predict = '95% PI',col.predict = 'black',xlab = "Effect size (95% CI)",
       colgap.forest = '1.5cm',colgap.studlab = '1cm',overall = F,overall.hetstat = F,prediction = F,fontfamily = 'sans',
       fs.heading = 16,fs.xlab = 16,ff.xlab = 2,ff.study = 3,ff.heading = 2,spacing = 1.2,addrow.subgroups = T,col.by = 'black' )

#openness
metr_o_std<-metagen(TE=estimate,seTE = se,data = meta_analysis_final_std_plt[65:78,],studlab = paste(Study),comb.fixed = F,comb.random = T,hakn = F,prediction =
 T,sm='SMD')
```

```r
metr_og_std<-update.meta(metr_o_std,byvar = Facet,comb.random = T,comb.fixed = F)
metr_og_std
forest(metr_og_std,layout = 'JAMA',text.predict = '95% PI',col.predict = 'black',xlab = "Effect size (95% CI)",colgap.forest = '1.5cm',colgap.studlab = '1cm')


#====================
# 5 Survival analysis
#====================
#libraries used are 'survival' and 'survminer'.
#functions used are 'survfit' and 'ggsurvplot'

library("survival")
library("survminer")
#pmi
colnames(blsa_survival)[which(names(blsa_survival)=="mort_compf")]<-"PMI"
b.cph.pmi<-survfit(Surv(time,status)~PMI,data=blsa_survival)
ggsurvplot(b.cph.pmi,pval = T,conf.int = F,risk.table = F, risk.table.col='strata',
           linetype = 'strata',surv.median.line = 'hv',ggtheme = theme_bw(),
           palette =  c("#EE799F", "#87CEEB", "#CDCD00", "#00868B","#2E9FDF"),
           font.x=c(20, 'bold'), font.y=c(20,'bold'), font.tickslab=c(18,'bold'),
           font.legend=c(16,'bold')
)

sard_cph$pim<-as.factor(sard_cph$mort_comp)
sard.cph.pim<-survfit(Surv(time,status)~pim,data=sard_cph)
ggsurvplot(sard.cph.pim,pval = T,conf.int = F,risk.table = F, risk.table.col='strata',
           linetype = 'strata',surv.median.line = 'hv',ggtheme = theme_bw(),
           palette =  c("#EE799F", "#87CEEB", "#CDCD00", "#00868B","#2E9FDF"),

)


#============================
#6 Causal Mediation Analysis
#============================
# lavaan package is used
# This package standardizes the estimates from the logistic and linear regressions
# before estimating the indirect effect so as to minimise the discrepancies
# between the a*b and c-c' estimates the method employed is described by
# Dawn Iacobucci (2012). Mediation analysis and categorical variables: The final frontier
# David P. MacKinnon and Matthew C. Cox (2012). Commentary on “Mediation Analysis and Categorical Variables:
# The Final Frontier” by Dawn Iacobucci
# The package uses structural equation modelling

library(lavaan)

####BLSA ####
model<-'
#Mediator
ave_mt_copy_number_30k ~ age + gender + a*mort_comp
#Mediator and predictor
deaths ~ age + gender + b*ave_mt_copy_number_30k + c*mort_comp

#indirect effect (a*b)
ie := a*b
#direct effect
de := c
#total effect
total := c + (a*b)
'
model.fit<-sem(model,data=blsa_neopir_final_pts,ordered = c('deaths'),
               se='bootstrap',bootstrap=5000,estimator='dwls')

summary(model.fit,fit.measures=T,rsquare=T,standardized=T)

####Sardinia #####
model2<-'
#Mediator
MT_count ~ Age + gender + a*mort_comp
#Mediator and predictor
deaths ~ Age + gender + b*MT_count + c*mort_comp

#indirect effect (a*b)
ie := a*b
#direct effect
de := c
#total effect
total := c + (a*b)
'
model2.fit<-sem(model2,data = wavedata1_plt,ordered = c('deaths'),
                se='bootstrap',bootstrap=5000,estimator='dwls')
summary(model2.fit,fit.measures=T,rsquare=T,standardized=T)
```
