# Supplementary material for: Personality traits are consistently associated with blood mitochondrial DNA copy number estimated from genome sequences in two genetic cohort studies
Source: eLife. 2022 Dec 20;11:e77806. doi: 10.7554/eLife.77806 (PMC9767459; doi:10.7554/eLife.77806)
Supplement: Transparent reporting form [file elife-77806-transrepform1.docx]

***eLife’s* transparent reporting form**

We encourage authors to provide detailed information *within their submission* to facilitate the interpretation and replication of experiments. Authors can upload supporting documentation to indicate the use of appropriate reporting guidelines for health-related research (see [EQUATOR Network](http://www.equator-network.org/%20)), life science research (see the [BioSharing Information Resource](https://biosharing.org/)), or the [ARRIVE guidelines](http://www.plosbiology.org/article/info:doi/10.1371/journal.pbio.1000412) for reporting work involving animal research. Where applicable, authors should refer to any relevant reporting standards documents in this form.

If you have any questions, please consult our Journal Policies and/or contact us: [editorial@elifesciences.org](mailto:editorial@elifesciences.org).

**Sample-size estimation**

- You should state whether an appropriate sample size was computed when the study was being designed
- You should state the statistical method of sample size computation and any required assumptions
- If no explicit power analysis was used, you should describe how you decided what sample (replicate) size (number) to use

Please outline where this information can be found within the submission (e.g., sections or figure legends), or explain why this information doesn’t apply to your submission:

The sample sizes in both cohorts were determined by the number of participants that had full whole-genome sequencing data because genome sequencing data were needed to estimate the mitochondrial DNA copy number (mtDNAcn). Details for sample sizes of the two cohorts are given in the subsection of the Materials and Methods entitled “Study cohorts’ description”.

**Replicates**

- You should report how often each experiment was performed
- You should include a definition of biological versus technical replication
- The data obtained should be provided and sufficient information should be provided to indicate the number of independent biological and/or technical replicates
- If you encountered any outliers, you should describe how these were handled
- Criteria for exclusion/inclusion of data should be clearly stated
- High-throughput sequence data should be uploaded before submission, with a private link for reviewers provided (these are available from both GEO and ArrayExpress)

Please outline where this information can be found within the submission (e.g., sections or figure legends), or explain why this information doesn’t apply to your submission:

This information doesn’t apply to our submission because we didn’t perform any biological experiment with replicates. We performed our analyses using already available data from two longitudinal aging cohorts.

Our inclusion/exclusion criteria are: for the BLSA cohort, we excluded non-white ancestry participants and participants who had been diagnosed with Alzheimer’s Disease; for the SardiNIA cohort, since it is a family cohort and mtDNAcn may be influenced by maternal and family DNA, we excluded one of each pair of participants with a genetic relationship above the level of second-degree cousins. Details of this are given in the subsection of the Materials and Methods entitled “Study cohorts’ description.”

**Statistical reporting**

- Statistical analysis methods should be described and justified
- Raw data should be presented in figures whenever informative to do so (typically when N per group is less than 10)
- For each experiment, you should identify the statistical tests used, exact values of N, definitions of center, methods of multiple test correction, and dispersion and precision measures (e.g., mean, median, SD, SEM, confidence intervals; and, for the major substantive results, a measure of effect size (e.g., Pearson's r, Cohen's d)
- Report exact p-values wherever possible alongside the summary statistics and 95% confidence intervals. These should be reported for all key questions and not only when the p-value is less than 0.05.

Please outline where this information can be found within the submission (e.g., sections or figure legends), or explain why this information doesn’t apply to your submission:

Statistical analysis methods were detailed in the “statistical analysis” subsection of the “Materials and Methods” section. Specifically, we used multiple linear regression to investigate the association between mtDNAcn (dependent variable) and personality traits (independent variables) while adjusting for known covariates. We reported associations as the number of standard deviations change in mtDNAcn in response to unit change of personality trait values with 95% confidence intervals (Figure 2). We further used a random-effect meta-analysis to obtain the combined estimates of effects and 95% confidence interval (CI) of each personality trait on mtDNAcn (Figure 2). We also reported all the regression effect sizes and p-values (both raw and FDR adjusted p-values) in Table 2 and Supplementary Table S2. Additionally, we used a Cox Proportional Hazards model to test the effect of personality on mortality (Figure 4A). Finally, we used a causal mediation analysis to test whether mtDNAcn mediates the association between personality and mortality risk. The effect estimates and p-values for this analysis were shown in Table 3 and Figure 4B.

(For large datasets, or papers with a very large number of statistical tests, you may upload a single table file with tests, Ns, etc., with reference to sections in the manuscript.)

**Group allocation**

- Indicate how samples were allocated into experimental groups (in the case of clinical studies, please specify allocation to treatment method); if randomization was used, please also state if restricted randomization was applied
- Indicate if masking was used during group allocation, data collection and/or data analysis

Please outline where this information can be found within the submission (e.g., sections or figure legends), or explain why this information doesn’t apply to your submission:

This information doesn’t apply to our submission because it is not a clinical study.

**Additional data files (“source data”)**

- We encourage you to upload relevant additional data files, such as numerical data that are represented as a graph in a figure, or as a summary table
- Where provided, these should be in the most useful format, and they can be uploaded as “Source data” files linked to a main figure or table
- Include model definition files including the full list of parameters used
- Include code used for data analysis (e.g., R, MatLab)
- Avoid stating that data files are “available upon request”

Please indicate the figures or tables for which source data files have been provided:

We have provided the R code used for all the data analyses performed in the manuscript.
